# Transgenic manipulation of triacylglycerol biosynthetic enzymes in *B. napus* alters lipid-associated gene expression and lipid metabolism

**DOI:** 10.1038/s41598-022-07387-x

**Published:** 2022-03-01

**Authors:** Pan Liao, Tamara Lechon, Trevor Romsdahl, Helen Woodfield, Stepan Fenyk, Tony Fawcett, Emma Wallington, Ruth E. Bates, Mee-Len Chye, Kent D. Chapman, John L. Harwood, Simon Scofield

**Affiliations:** 1 School of Biological Science, University of Hong Kong, Pokfulam, Hong Kong; 2 School of Biosciences, Cardiff University, Cardiff, CF10 3AX UK; 3 Department of Biosciences, Durham University, Durham, UK; 4 John Bingham Lab., National Institute for Agricultural Botany, Cambridge, CB3 0LE UK; 5 Department of Biological Sciences, BioDiscovery Institute, University of North Texas, Denton, TX 76203-5017 USA

**Keywords:** Biochemistry, Plant sciences

## Abstract

Oilseed rape (*Brassica napus*) is an important crop that is cultivated for the oil (mainly triacylglycerol; TAG) it produces in its seeds. TAG synthesis is controlled mainly by key enzymes in the Kennedy pathway, such as glycerol 3-phosphate acyltransferase (GPAT), lysophosphatidate acyltransferase (LPAT) and diacylglycerol acyltransferase (DGAT) but can also be produced from phosphoglycerides such as phosphatidylcholine (PC) by the activity of the enzyme phospholipid: diacylglycerol acyltransferase (PDAT). To evaluate the potential for these enzymes to alter oil yields or composition, we analysed transgenic *B. napus* lines which overexpressed GPAT, LPAT or PDAT using heterologous transgenes from Arabidopsis and Nasturtium and examined lipid profiles and changes in gene expression in these lines compared to WT. Distinct changes in PC and TAG abundance and spatial distribution in embryonic tissues were observed in some of the transgenic lines, together with altered expression of genes involved generally in acyl-lipid metabolism. Overall our results show that up-regulation of these key enzymes differentially affects lipid composition and distribution as well as lipid-associated gene expression, providing important information which could be used to improve crop properties by metabolic engineering.

## Introduction

Oilseed rape (*Brassica napus*) is the world’s third most important oil crop and contributes about 16% of total plant oil production^1^. There are two distinct types of oilseed rape which are the high-erucic acid (HEAR) and low-erucic acid (LEAR) containing varieties. The former is used mainly as an industrial feedstock while the LEAR varieties are used for human food and animal feeds^2,3^. *B. napus* can be genetically modified relatively easily and much North American production is of transgenic crop varieties. Moreover, *B. napus* is closely related to *Arabidopsis thaliana*^4^ so that knowledge of this model species can often assist development of improved *B. napus* lines.

Oil accumulation in *B. napus* is catalysed mainly by the Kennedy pathway. Detailed studies using flux control analysis showed that lipid assembly by the Kennedy pathway exerted strong control over triacylglycerol (TAG) biosynthesis and, hence, oil accumulation^5,6^. Earlier biochemical experiments suggested that, of the four enzymes in the Kennedy pathway, the final enzyme, termed diacylglycerol acyltransferase (DGAT) was particularly important^7,8^. Indeed, increasing *DGAT1* gene expression altered flux control of TAG synthesis significantly^3^ and led to increased oil yields in field trials^9^.

Of the four enzymes in the Kennedy pathway for TAG biosynthesis, the first is glycerol 3-phosphate acyltransferase (GPAT). In Arabidopsis, there are nine isoforms, of which the GPAT9 clade is responsible for TAG formation^10–12^. A summary of data published on the characteristics of the Arabidopsis genes (plastid and endoplasmic reticulum) and GPAT functions are found in Ref.^12^. The *GPAT9* gene is expressed significantly during embryogenesis^13^ and also contributes to oil droplet formation in developing pollen grains^11^. Expression of two heterologous genes for GPAT in Arabidopsis produced small increases in TAG accumulation^14^ while efforts to boost supply of its substrate in *B. napus* increased oil formation^15,16^.

The second enzyme in the Kennedy pathway, lysophosphatidate/lysophosphatidic acid acyltransferase (LPAT/LPAAT) belongs to the MBOAT (membrane-bound *O*-acyltransferase) superfamily. There are two main groups in plants. The first group is plastidial and termed LPAAT1. The second group (which is closely related to various animal, yeast or prokaryotic LPAATs) is termed LPAAT2-5. In plants, LPAAT activity has been reported in various organelles or membrane fractions^12^. They usually have greater substrate selectivity than other Kennedy pathway enzymes^17,18^. This has led to particular plant LPAATs being used to manipulate the final fatty acid content of plant oils^19^ and many manipulations have resulted in higher seed oil contents^20–23^. We examined the effect of LPAAT overexpression in *B. napus* in relation to flux control and concluded that the increase in oil occurred despite a low intrinsic flux control coefficient^24^.

The final Kennedy pathway enzyme, DGAT, normally catalyses the formation of TAG from diacylglycerol. However, a second enzyme, termed phospholipid: diacylglycerol acyltransferase (PDAT), is capable of esterifying diacylglycerol in an acyl-CoA independent reaction. PDAT was reported in plants^25^ and characterised further^26^. The relative contribution of DGAT and PDAT to oil accumulation in plants varies with species and is a matter of some discussion^27^, though evidence from *B. napus*^28^ and Arabidopsis^29^ indicates that DGAT is more important, although these enzymes have overlapping functions in Arabidopsis seed development^30^.

Since we had previously shown that lipid assembly in *B. napus* was important for regulation of oil accumulation^3,6^, in this study we examined lipid profiles and changes in global gene expression in transgenic *B. napus* lines overexpressing three important enzymes for triacylglycerol formation: glycerol-3-phosphate acyltransferase 9 (GPAT9), lysophosphatidate acyltransferase (LPAT/LPAAT) and phospholipid:diacylglycerol acyltransferase (PDAT). We found distinct changes in accumulation and tissue-specific distribution of phosphatidylcholine and TAG lipid species in the embryos of the transgenic lines compared to wild-type (WT) control and correlated this with changes in the expression of genes encoding proteins with functions associated with lipid and fatty acid metabolism. Such changes provide important information for future research with one of the world’s most important oil crops.

## Results

### Characterisation of *B. napus* transgenic lines during development

**Figure 1:**
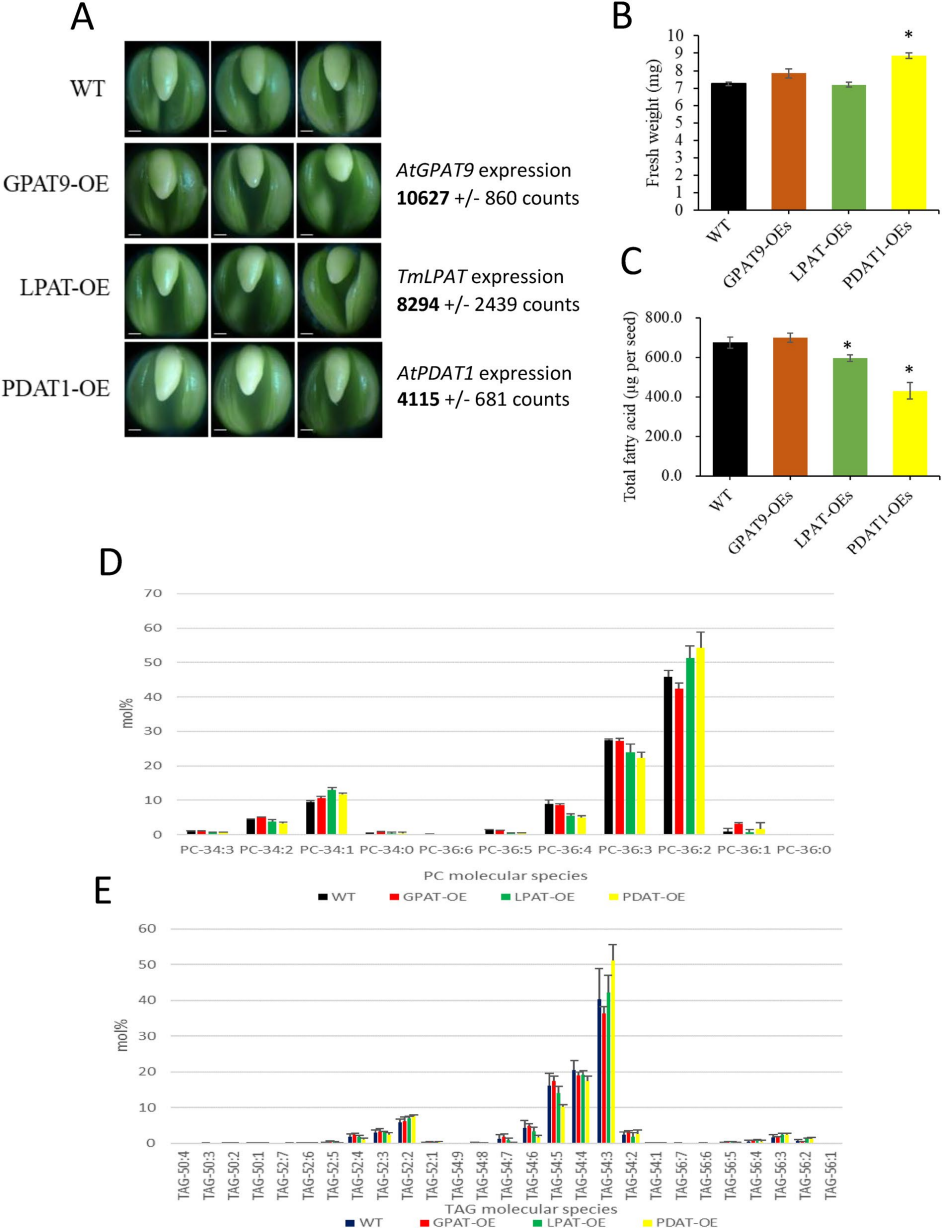
Analyses on morphology, fresh weight and total fatty acid content on embryos or seeds at mid-developmental stage. (**A**) Photos of embryos at middle stage, 38 days after pollination (DAP) in Hong Kong. Scale bar = 200 μm. RNA-seq normalized counts for the respective transgenes are shown to the right of the images. Transgene expression was not detected in WT samples, except for one read for *AtGPAT9* which mapped to an endogenous *B. napus* GPAT in the WT sample. (**B**) Fresh seed weight at 38 DAP. Values are means ± S.E. (*n* = 17–45). (**C**) Total fatty acid (FA) content on the basis of seed number at 38 DAP. *Indicates significant differences (*P* < 0.05) from wild type (DH12075) by Student’s *t* test. (**D**) Relative abundance (mol%) of PC molecular species and (**E**) TAG molecular species determined by MALDI-MS in fully mature whole embryos.

The three homozygous transgenic lines analysed in this study overexpressed transgenes encoding glycerol 3-phosphate acyltransferase 9 (*GPAT9*) from *Arabidopsis thaliana* (GPAT-OE), lysophosphatidic acid acyltransferase from *Tropaeolum majus* (LPAT-OE) and phospholipid: diacylglycerol acyltransferase1 from *Arabidopsis thaliana* (PDAT-OE). These represent the first two steps of the Kennedy pathway and the acyl-CoA-free formation of triacylglycerol (TAG), respectively. Transgene expression in each line was measured by RNA-seq analysis (Fig. 1A) and confirmed high-level expression as shown by normalised counts. A comparison of seed weight, embryo morphology and total fatty acid content in the transgenic lines in relation to the wild-type control at the mid-stage of development, which correlates with the phase of rapid oil accumulation, is shown in Fig. 1. While no significant alteration in embryo morphology was observed compared to WT (Fig. 1A), there was a small significant increase in fresh seed weight in PDAT-OE but not in GPAT-OE or LPAT-OE (Fig. 1B) at this developmental stage. Furthermore, both LPAT-OE and PDAT-OE accumulated significantly less total fatty acid per seed, with reductions of 12% and 36%, respectively, while there were no significant alterations in the GPAT-OE (Fig. 1C).

### MALDI-MS analysis of PC and TAG molecular species quantification and spatial distribution

Our observations indicated some changes in fatty acid and hence acyl lipid metabolism, therefore we compared seeds of the three transgenic lines with controls using MALDI-MS imaging to visualise the spatial distributions of phosphatidylcholine (PC) and triacylglycerol (TAG) molecular species in mature embryos. We had employed this method in *B. napus* previously to examine lipid metabolism during seed development^31^. Molecular species of PC were measured because this phosphoglyceride is particularly important as a metabolic intermediate during oil accumulation^27^.

Although not suitable for absolute quantification, MALDI-MS does allow for the relative quantification of molecular species within a given lipid class, and this approach has been validated in *B. napus* seeds elsewhere by additional quantitative MS^31^. The relative quantification of molecular species of PC and TAG in mature whole embryos is shown in Fig. 1D,E and Supplementary Table S1. As expected for a high oleate seed and consistent with previous evidence^31^, the major species of PC in WT samples was 36:2 (dioleoyl; 47%) followed by 36:3 (27%), 34:1 (10%), 36:4 (9%) and 34:2 (4%). All other measured PC species constituted 1% or less of the total PC content. The predominant TAG species in WT was 54:3 (trioleoyl; 40%), followed by 54:4 (21%), 54:5 (16%), 52:2 (6%), 54:6 (4%), 52:3 (3%), 52:4 (2%) and 56:3 (2%), with all other measured TAG species constituting ~ 1% or less of total TAG content.

There was very little change in the relative abundance of the predominant PC species in the GPAT-OE line compared to WT, except for an increase in 36:1 content from 1 to 3% and a small decrease in 36:2. However, in LPAT-OE and PDAT-OE lines there was an increase in the relative abundance of PC 36:2 and a small decrease in the amount of 36:3 and 36:4, together with a minor increase in 34:1. TAG species 54:3 showed little change in GPAT-OE and LPAT-OE samples but showed an increase in PDAT-OE (though this failed to meet the threshold for statistical significance due to the relatively large standard deviation in the WT sample) together with a small decrease in 54:4, 54:5 and 54:6 levels in PDAT-OE relative to WT.

**Figure 2:**
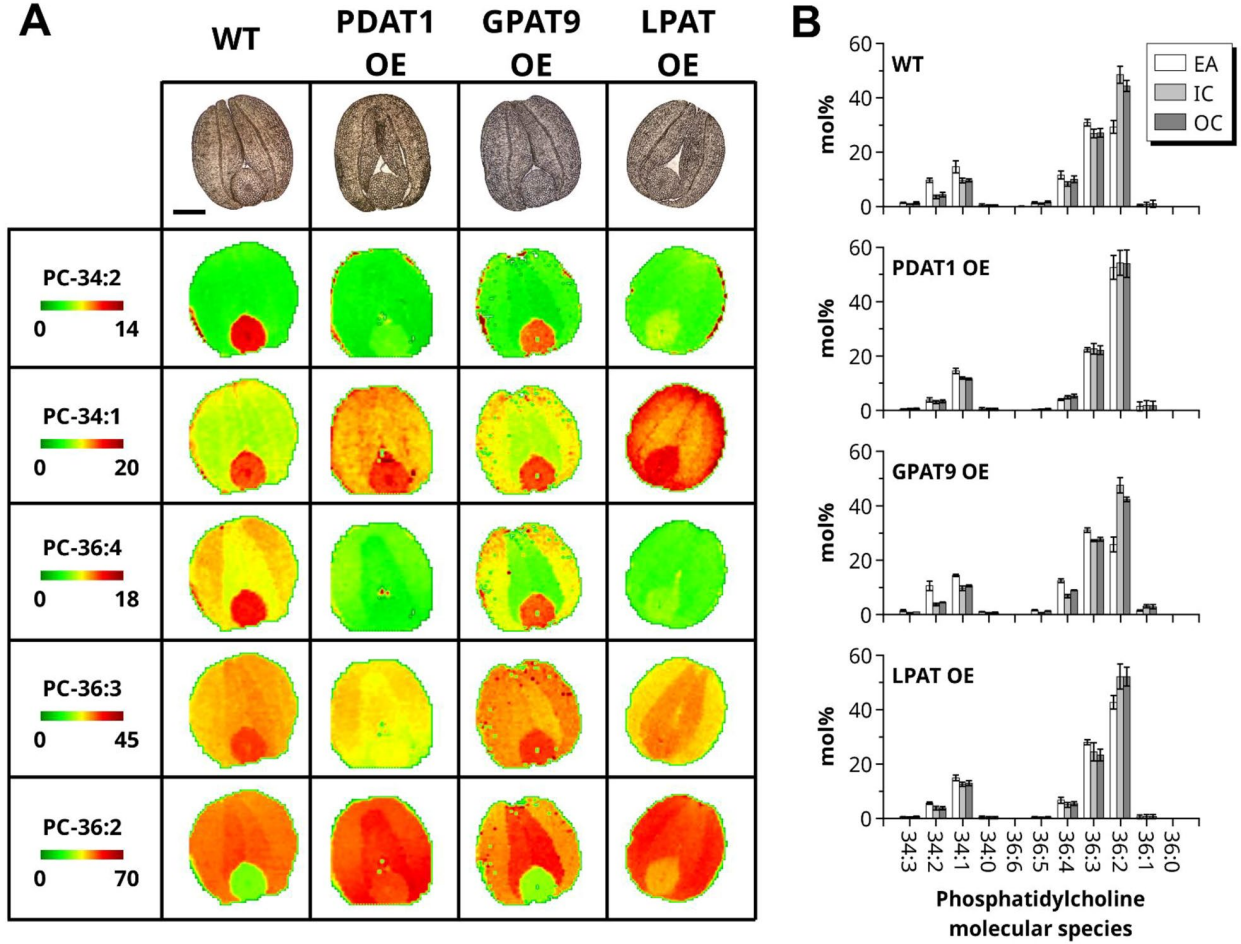
MALDI-MS imaging of PC in mature embryos from the WT and transgenic lines. (**A**) MS imaging of selected PC molecular species for WT (DH12075), PDAT1 OE, GPAT9 OE, and LPAT OE. Molecular species are scaled at different intensities to highlight tissue specific distributions on a green to red color scale, indicated in the first column. Bright-field images of the tissue sections used in MS imaging experiments are shown in the first row for each genotype with the embryonic axis located at the bottom (scale bar = 500 μm). (**B**) Average mol% of PC molecular species for each genotype summed over the tissue surface areas; embryonic axis (EA, white), inner cotyledons (IC, light gray), and outer cotyledons (OC, dark gray). (n = 4, ± SD).

**Figure 3:**
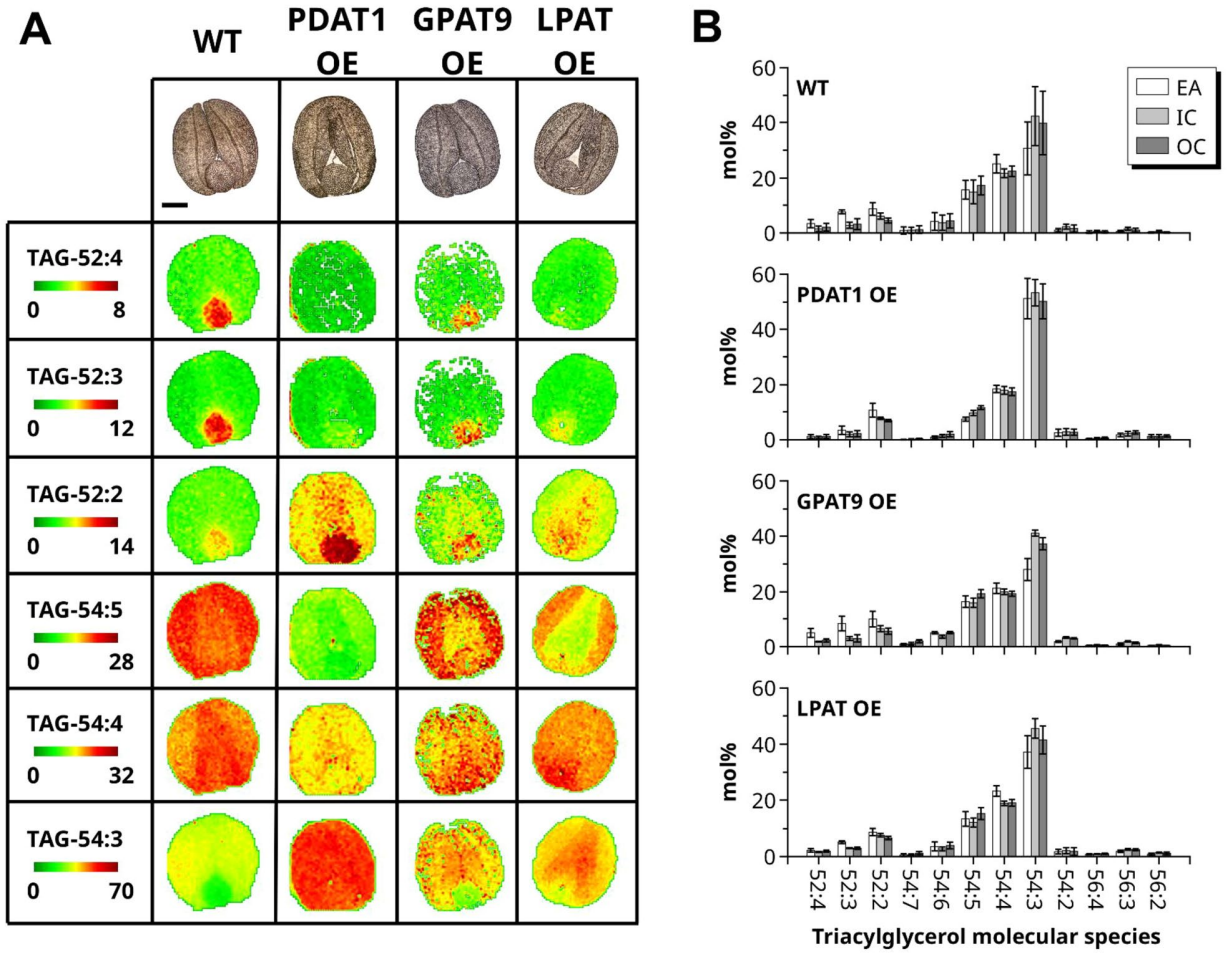
MALDI-MS imaging of TAG mature embryos from the WT and transgenic lines. (**A**) MS imaging of selected TAG molecular species for WT (DH12075), PDAT1 OE, GPAT9 OE, and LPAT OE. Molecular species are scaled at different intensities to highlight tissue specific distributions on a green to red color scale, indicated in the first column. Bright-field images of the tissue sections used in MS imaging experiments are shown in the first row for each genotype with the embryonic axis located at the bottom (scale bar = 500 μm). (**B**) Average mol% of TAG molecular species for each genotype summed over the tissue surface areas; embryonic axis (EA, white), inner cotyledons (IC, light gray), and outer cotyledons (OC, dark gray). (n = 4, ± SD).

We next analysed the spatial distribution of the different PC and TAG molecular species using MALDI-MS imaging of embryos (Figs. 2 and 3; Supplementary Fig. S1 and Fig. S2). The percentage of each molecular species was relatively quantified in the embryonic axis (hypocotyl, shoot and root poles) and the inner- and outer-cotyledons of WT embryos and those of the three transgenic lines and is shown in the bar charts of Figs. 2B and 3B and represented by heat-map colour intensity (green low, red high) on the MALDI-MS images. The WT and GPAT-OE MALDI-MS images for PC distribution were similar (Fig. 2A,B). For example, the 34:2 species was predominantly concentrated in the embryonic axis in both of these genotypes, whereas the 36:2 species was lower in this region of the embryo relative to the cotyledons. Such a distinct heterologous distribution was not apparent in the LPAT-OE or PDAT-OE lines, with lower levels of 34:2, 36:3 and 36:4 and higher levels of 36:2 in the embryonic axis of these lines leading to a more homogenous distribution throughout the embryo.

For TAG, the MALDI-MS image data for WT and GPAT-OE were again broadly similar (Fig. 3A,B), with 52:2, 52:3 and 52:4 species showing predominant accumulation in the embryonic axis compared to cotyledons, while 54:4 and 54:5 were detected at higher levels throughout the embryo. TAG 54:3 showed homogenous accumulation in the WT sample with similar levels in cotyledons in GPAT-OE. By contrast, LPAT-OE and PDAT-OE showed reduced accumulation of 52:4 and 52:3 in the embryonic axis compared to WT. Moreover, there was a strong increase in 52:2 and 54:3 species in PDAT-OE in the embryonic axis and cotyledons and a moderate increase in these species in the same tissues of LPAT-OE. For 54:4 and 54:5 species, there were lower relative levels in PDAT-OE and only minor reductions in LPAT-OE and GPAT-OE.

In summary, GPAT overexpression has little effect on PC and TAG molecular species distribution in the embryonic tissues, while LPAT and PDAT overexpression reduced the distinct heterologous distribution. Overall, these results show that overexpression of key enzymes for TAG biosynthesis can have substantial effects on the usual pattern of oil accumulation found in seeds on high-oleic *B. napus*.

### Transcriptomic analysis of GPAT-OE, LPAT-OE and PDAT-OE transgenic lines

**Figure 4:**
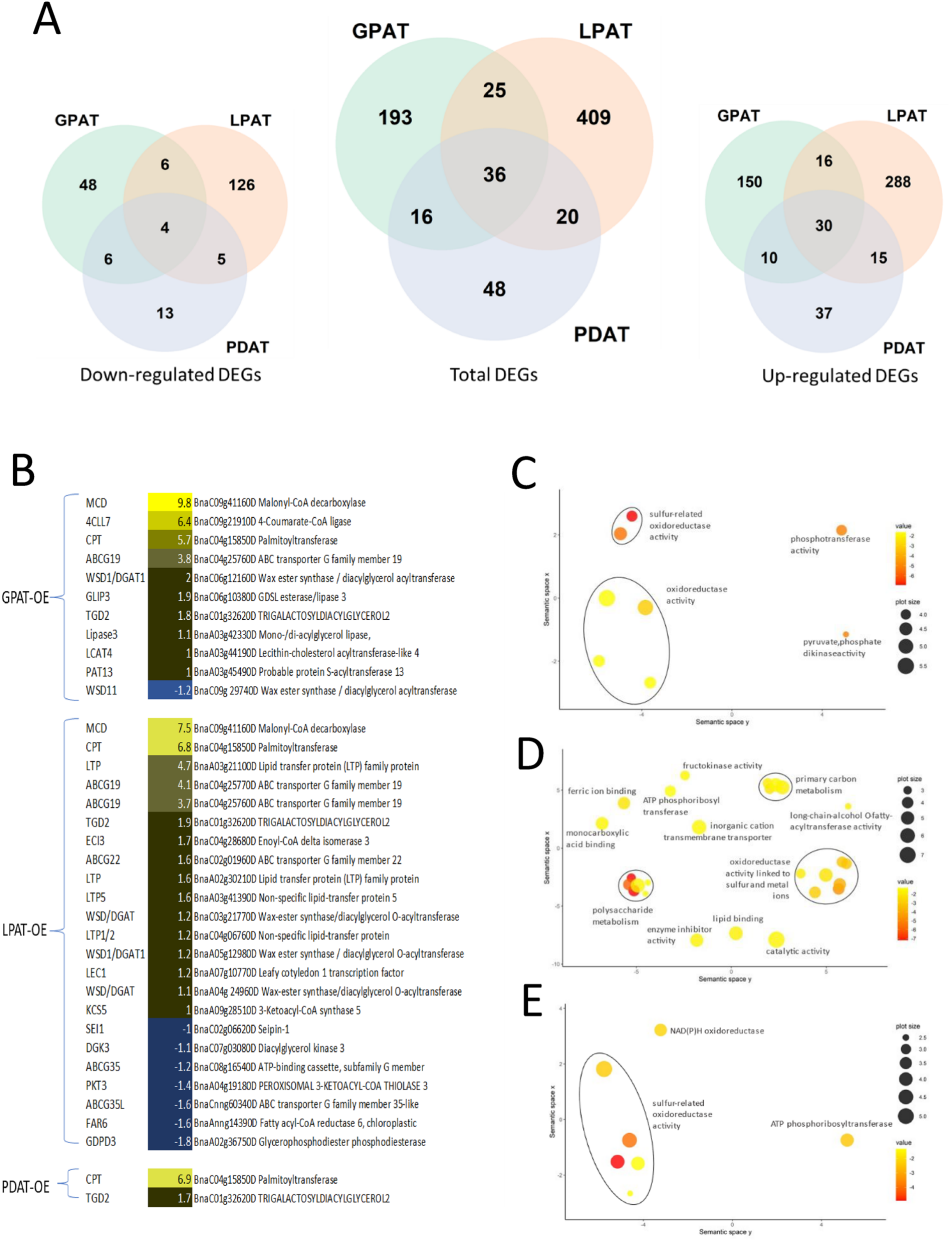
Transcriptomic analysis of GPAT9-OE, LPAT-OE and PDAT-OE transgenic lines. (**A**) Total number of differentially expressed genes (DEGs) identified in comparisons of GPAT9, PDAT or LPAT overexpressing lines compared to WT control. (**B**) Heat-map of selected DEGs related to lipid metabolism. (**C**–**E**) GO enrichment analysis for molecular function in the GPAT-OE (**C**), LPAT-OE (**D**) and PDAT-OE (**E**) lines.

We carried out a detailed transcriptomic analysis by RNA-seq on embryos of WT and the transgenic lines at the mid-stage of seed development, which corresponds to the phase of rapid oil accumulation in *B. napus*^31^. We compared the mRNA transcript profiles for the WT, GPAT-OE, LPAT-OE and PDAT-OE and identified differentially expressed genes (DEGs) between the WT and transgenic lines (Fig. 4A) by applying 0.01% statistical significance and twofold expression change filtering criteria for differential expression. In total we detected 270 DEGs in the GPAT-OE sample compared to WT, 490 DEGs in LPAT-OE compared to WT and 120 DEGs in PDAT-OE compared to WT (Supplementary Table S2 for full list of differentially expressed genes), with substantially more genes displaying up-regulation compared to down-regulation in all samples.

We performed gene ontology (GO) enrichment analysis on the sets of DEGs and revealed enrichment of specific classes of molecular function in each of the transgenic lines. Notably, GPAT-OE lines showed enrichment of genes associated with oxidoreductase activity, phosphotransferase and kinase activity, PDAT-OE lines showed enrichment of oxidoreductase and phosphoribosyl transferase activity, while LPAT-OE lines showed enrichment of a broader range of molecular functions, including fatty-acyl transferase activity, lipid binding, oxidoreductase activity, polysaccharide metabolism, primary carbon metabolism, transferase and transporter activity (Fig. 4C–E).

We then examined the DEGs for each transgenic line to identify genes associated with acyl-lipid metabolism (Fig. 4B; Table 1). Among the 36 genes which showed differential expression in all three transgenic lines, two genes were identified with roles in acyl-lipid metabolism: a putative cysteine *S*-palmitoyltransferase (*CPT*), which is used for lipid modification of proteins, and *TRIGALACTOSYLDIACYLGLYCEROL2* (*TGD2*), which is involved in lipid transfer from ER to chloroplast. For GPAT-OE and LPAAT-OE lines, malonyl-CoA decarboxylase (*MCD*) was notably up-regulated. For LPAT-OE lines, putative ABC transporters, various lipid transport proteins and wax ester synthase/diacylglycerol *O*-acyltransferases (WES/DGAT) were up-regulated while seipin-1, diacylglycerol kinase and enzymes for fatty acid oxidation were down-regulated. In GPAT-OE, an ABC transporter and lipases were up-regulated while one WES/DGAT was up-regulated with another down-regulated. In PDAT-OE only *CPT* and *TGD2* were directly relevant to lipid biochemistry (Table 1; Fig. 4B).

**Table 1:** Expression values (normalised counts) of selected DEGs associated with acyl-lipid metabolism and related enzymatic functions in GPAT-OE (A), LPAT-OE (B) and PDAT-OE (C) lines compared to WT. Values highlighted in italics and underline indicate significant up-regulation and down-regulation respectively.

Gene	ID	Kegg orthology	Abbreviation	FoldChange	Log2FoldChange	Count (WT)	Count (GPAT-OE)	Count (LPAT-OE)	Count (PDAT-OE)
**(A) Selected DEGs in GPAT-OE versus WT**									
BnaC09g41160D	GSBRNA2T00094930001	**Malonyl-CoA decarboxylase**	MCD	902.57	9.82	1	*1055 *	*189 *	5
BnaC09g21910D	GSBRNA2T00067333001	**4-Coumarate-CoA ligase**	4CLL7	90.10	6.49	0	*20 *	1	0
BnaC04g15850D	GSBRNA2T00089970001	**Palmitoyltransferase**	CPT	52.36	5.71	0	*11 *	*23 *	*26 *
BnaC04g25760D	GSBRNA2T00115151001	**ABC transporter G family member 19**	ABCG19	14.18	3.83	39	*669 *	*581 *	8
BnaA02g00090D	GSBRNA2T00143567001	Soluble inorganic pyrophosphatase 6, chloroplastic	PPA6	6.36	2.67	8	*62 *	14	12
BnaC01g26830D	GSBRNA2T00001735001	NADPH-dependent aldehyde reductase 1, chloroplastic	ChlADR1	5.73	2.52	30	*201 *	5	30
BnaA01g18450D	GSBRNA2T00021277001	**Pyruvate, phosphate dikinase 1, chloroplastic **	PPDK	5.70	2.51	5	*31 *	6	*14 *
BnaC01g27670D	GSBRNA2T00086312001	ATP phosphoribosyltransferase 1, chloroplastic	ATP-PRT1	5.69	2.51	27	*179 *	*147 *	*228 *
BnaA08g12320D	GSBRNA2T00021894001	Protein phosphatase 2C	PP2C	5.56	2.48	6	*37 *	12	7
BnaA02g21620D	GSBRNA2T00087431001	Dehydrodolichyl diphosphate synthase 5	DHDDS	4.15	2.05	13	*65 *	8	15
BnaC01g36750D	GSBRNA2T00051881001	Terpenoid synthase 25	TPS25	4.11	2.04	8	*40 *	3	13
BnaC06g12160D	GSBRNA2T00036788001	**Wax ester synthase/diacylglycerol acyltransferase**	WSD1/DGAT1	4.01	2.00	5	*23 *	*14 *	*16 *
BnaC06g10380D	GSBRNA2T00045998001	**GDSL esterase/lipase 3**	GLIP3	3.94	1.98	19	*88 *	15	33
BnaC01g32620D	GSBRNA2T00095870001	**TRIGALACTOSYLDIACYLGLYCEROL2**	TGD2	3.68	1.88	8	*33 *	*31 *	*30 *
BnaA02g30810D	GSBRNA2T00055414001	FT-interacting protein 3	FTIP3	3.29	1.72	8	*31 *	*305 *	*50 *
BnaA05g24350D	GSBRNA2T00104876001	Protein phosphatase 2C, putative	PP2C	2.34	1.23	22	*61 *	16	25
BnaC07g33060D	GSBRNA2T00031454001	**Pyruvate, phosphate dikinase 1, chloroplastic**	PPDK	2.19	1.13	291	*741 *	268	369
BnaA03g42330D	GSBRNA2T00151180001	**Mono-/di-acylglycerol lipase, N-terminal;Lipase, class 3 **	Lipase3	2.14	1.10	64	*161 *	74	73
BnaA03g44190D	GSBRNA2T00106681001	**Lecithin-cholesterol acyltransferase-like 4**	LCAT4	2.11	1.08	62	*151 *	62	79
BnaA03g45490D	GSBRNA2T00106844001	**Probable protein S-acyltransferase 13**	PAT13	2.11	1.07	43	*106 *	49	52
BnaC07g34140D	GSBRNA2T00031293001	Ankyrin repeat family protein	ANK	2.10	1.07	218	*534 *	192	248
BnaA03g42800D	GSBRNA2T00151123001	*O*-fucosyltransferase 29	OFUT29	2.07	1.05	72	*172 *	82	95
BnaA09g46120D	GSBRNA2T00055232001	Probable inactive purple acid phosphatase 1	PAP1	2.06	1.04	31	*75 *	33	46
BnaC09g29740D	GSBRNA2T00059030001	**Wax ester synthase/diacylglycerol acyltransferase**	WSD11	− 2.38	− 1.25	424	205	229	521
**(B) Selected DEGs in LPAT-OE versus WT**									
BnaC09g41160D	GSBRNA2T00094930001	**Malonyl-CoA decarboxylase**	MCD	185.05	7.53	1	*1055 *	*189 *	5
BnaC04g15850D	GSBRNA2T00089970001	**Palmitoyltransferase**	CPT	118.52	6.89	0	*11 *	*23 *	*26 *
BnaA03g21100D	GSBRNA2T00138964001	**Lipid transfer protein (LTP) family protein **	LTP	27.39	4.78	1	*8 *	*28 *	*5 *
BnaAnng13920D	GSBRNA2T00017498001	Pyruvate decarboxylase	PDC	27.17	4.76	1	2	*19 *	3
BnaC04g25770D	GSBRNA2T00115147001	**ABC transporter G family member 19**	ABCG19	18.00	4.17	14	*340 *	*267 *	4
BnaC04g25760D	GSBRNA2T00115151001	**ABC transporter G family member 19**	ABCG19	13.69	3.78	39	*669 *	*581 *	8
BnaA02g15870D	GSBRNA2T00116433001	Basic helix-loop-helix (bHLH) family protein	BHLH96	11.34	3.50	2	3	*20 *	4
BnaC08g15350D	GSBRNA2T00047383001	**EXTENSIN-LIKE PROTEIN**	ELP	10.93	3.45	30	33	*342 *	53
BnaC01g08820D	GSBRNA2T00123018001	Alkaline-phosphatase-like family protein		10.54	3.40	4	0	*45*	3
BnaA05g15620D	GSBRNA2T00043349001	Cellulose synthase A	CESA	8.85	3.15	10	*95 *	*96 *	*110 *
BnaA05g33500D	GSBRNA2T00022315001	Plant stearoyl-acyl-carrier desaturase family protein	S-ACP-DES	7.96	2.99	2	5	*17 *	4
BnaA02g11230D	GSBRNA2T00019888001	Trehalose 6-phosphate phosphatase	TPPA	7.09	2.83	5	7	*35 *	*17 *
BnaA04g27920D	GSBRNA2T00007060001	Ribulose bisphosphate carboxylase small chain 2B	RBCS-2B	5.71	2.51	16	27	*95 *	33
BnaA01g33320D	GSBRNA2T00057206001	Starch synthase, chloroplastic/amyloplastic	SS2	4.25	2.09	45	42	*200 *	*493 *
BnaCnng03960D	GSBRNA2T00032432001	Pyruvate decarboxylase 1	PDC1	3.78	1.92	99	*215 *	*391 *	157
BnaC01g32620D	GSBRNA2T00095870001	**TRIGALACTOSYLDIACYLGLYCEROL2**	TGD2	3.68	1.88	8	*33 *	*31 *	*30 *
BnaC04g28680D	GSBRNA2T00070367001	**Enoyl-CoA delta isomerase 3**	ECI3	3.44	1.78	9	16	*33 *	17
BnaC02g01960D	GSBRNA2T00078617001	**ABC transporter G family member 22**	ABCG22	3.24	1.69	17	15	*58 *	21
BnaA10g10940D	GSBRNA2T00052811001	GATA transcription factor 21	GATA21	3.24	1.69	14	9	*47 *	13
BnaA02g30210D	GSBRNA2T00091667001	**Lipid transfer protein (LTP) family protein **	LTP	3.22	1.69	20	29	*67 *	28
BnaC02g03770D	GSBRNA2T00152038001	Acyl-CoA N-acyltransferases (NAT) superfamily protein	GNAT	3.18	1.67	7	12	*25 *	9
BnaCnng66200D	GSBRNA2T00093985001	Transcription factor bHLH93	BHLH93	3.11	1.64	84	87	*271 *	146
BnaA03g41390D	GSBRNA2T00151289001	**Non-specific lipid-transfer protein 5 **	LTP5	3.11	1.64	20	*51 *	*66 *	39
BnaA06g13060D	GSBRNA2T00035619001	Isochorismate synthase 2, chloroplastic	ICS2	2.52	1.33	70	93	*186 *	97
BnaC04g34390D	GSBRNA2T00108243001	Ribulose bisphosphate carboxylase small chain 1B	RBCS-1B	2.48	1.31	63	49	*162 *	70
BnaC03g21770D	GSBRNA2T00079085001	**Wax-ester synthase/diacylglycerol *O*-acyltransferase**	WSD/DGAT	2.41	1.27	27	24	*68 *	32
BnaC04g06760D	GSBRNA2T00152824001	**Non-specific lipid-transfer protein**	LTP1/2	2.36	1.24	97	169	*238 *	145
BnaA05g12980D	GSBRNA2T00066242001	**Wax-ester synthase/diacylglycerol *O*-acyltransferase**	WSD1/DGAT1	2.35	1.23	53	*126 *	*128 *	91
BnaA07g10770D	GSBRNA2T00112245001	**Leafy cotyledon 1 transcription factor**	LEC1	2.35	1.23	49	64	*120 *	59
BnaA04g24960D	GSBRNA2T00045091001	**Wax-ester synthase/diacylglycerol *O*-acyltransferase**	WSD/DGAT	2.26	1.18	16	30	*39 *	24
BnaA09g28510D	GSBRNA2T00074279001	**3-Ketoacyl-CoA synthase 5**	KCS5	2.13	1.09	53	53	*119 *	67
BnaC05g20500D	GSBRNA2T00142427001	Phosphoenolpyruvate carboxylase	PEPC	2.12	1.08	27	54	60	26
BnaA04g08570D	GSBRNA2T00114447001	Ribulose bisphosphate carboxylase small chain	RBCS	2.09	1.06	78	112	*170 *	118
BnaC01g00830D	GSBRNA2T00131222001	Phospho-2-dehydro-3-deoxyheptonate aldolase 1	DHS1	2.04	1.03	485	598	*1038 *	650
BnaC03g13590D	GSBRNA2T00060779001	Pyruvate kinase	PK	2.02	1.02	131	147	*280 *	149
BnaC02g06620D	GSBRNA2T00065388001	**Seipin-1**	SEI1	− 2.03	− 1.02	358	487	184	433
BnaC03g15880D	GSBRNA2T00052649001	Protein phosphatase 2C, putative	PP2C	− 2.12	− 1.08	110	181	55	112
BnaC07g03080D	GSBRNA2T00070799001	**Diacylglycerol kinase 3**	DGK3	− 2.20	− 1.14	96	121	46	80
BnaA09g00710D	GSBRNA2T00018414001	Sucrose synthase	SUS3	− 2.40	− 1.26	867	1218	381	992
BnaC08g16540D	GSBRNA2T00139785001	**ATP-binding cassette, subfamily G (WHITE), member 2**	ABCG35	− 2.43	− 1.28	108	209	46	91
BnaAnng30500D	GSBRNA2T00088106001	Inorganic pyrophosphatase 1	PS2	− 2.49	− 1.31	83	*199 *	35	98
BnaA04g19180D	GSBRNA2T00064654001	**PEROXISOMAL 3-KETOACYL-COA THIOLASE 3**	PKT3	− 2.63	− 1.40	37	56	15	50
BnaA09g32020D	GSBRNA2T00129280001	Protein phosphatase 2C, putative	PP2C	− 2.68	− 1.42	38	28	15	45
BnaCnng60340D	GSBRNA2T00077579001	**ABC transporter G family member 35-like**	ABCG35L	− 3.04	− 1.60	53	100	18	46
BnaAnng14390D	GSBRNA2T00020066001	**Fatty acyl-CoA reductase 6, chloroplastic**	FAR6	− 3.15	− 1.66	785	1544	263	1107
BnaA02g36750D	GSBRNA2T00084092001	**Glycerophosphodiester phosphodiesterase**	GDPD3	− 3.65	− 1.87	26	*58 *	7	18
**(C) Selected DEGs in PDAT-OE versus WT**									
BnaC04g15850D	GSBRNA2T00089970001	**Palmitoyltransferase**	CPT	125.49	6.97	0	*11 *	*23 *	*26 *
BnaA01g33320D	GSBRNA2T00057206001	**Starch synthase, chloroplastic/amyloplastic **	SS2	9.81	3.30	45	42	*200 *	*493 *
BnaA05g15620D	GSBRNA2T00043349001	**Cellulose synthase A**	CESA	9.51	3.25	10	*95 *	*96 *	*110 *
BnaC01g27670D	GSBRNA2T00086312001	ATP phosphoribosyltransferase	ATP-PRT	7.65	2.94	27	*179 *	*147 *	*228 *
BnaA01g21560D	GSBRNA2T00024207001	ATP phosphoribosyltransferase	ATP-PRT	4.35	2.12	34	*66 *	*106 *	*169 *
BnaC01g32620D	GSBRNA2T00095870001	**TRIGALACTOSYLDIACYLGLYCEROL2**	TGD2	3.68	1.88	8	*33 *	*31 *	*30 *
BnaC08g14300D	GSBRNA2T00012806001	RNA cytidine acetyltransferase 1	RRA1	− 2.22	− 1.15	126	142	203	64
BnaCnng62170D	GSBRNA2T00083507001	Starch synthase	SS	− 100.00	− 6.64	17	2	0	0

Bold indicates genes directly related to acyl-lipid metabolism.

Because of the importance of plant acyl-CoA binding proteins (ACBPs) during seed development and oil accumulation^32^, we examined *ACBP* expression in embryos from developing *B. napus* seeds in the three transgenic lines compared to WT (Supplementary Fig. S3). Previous studies on *B. napus* gene expression provided background information about the relative expression of the six *ACBPs* (*ACBP1*–*ACBP6*) and their isoforms^33^. No significant differential expression of *ACBPs* was detected by RNA-seq analysis in any of the transgenic lines, but there was a decrease in *ACBP2* expression in the LPAT-OE line that just failed to meet the differential expression threshold, indicating possible reduced activity of ACBP2 in the LPAT-OE line.

## Discussion

In this paper, we studied the effect of overexpression of genes encoding three important enzymes for triacylglycerol formation, and hence oil accumulation, in *Brassica napus*. GPAT catalyses the first step of the Kennedy pathway and expression of two heterologous GPAT genes in Arabidopsis has been shown previously to produce modest increases in oil content^14^. In addition, other studies^15,16^ have shown that boosting the supply of GPAT’s substrate, glycerol 3-phosphate, can increase TAG accumulation in oilseed rape. There are several isoforms of GPAT in plants and analysis of the gene family reveals that the *GPAT9* clade is responsible for TAG biosynthesis and oil accumulation^12^, and these functions have been demonstrated unequivocally in Arabidopsis by two separate groups^10,11^.

We overexpressed Arabidopsis *GPAT9* in oilseed rape and found no effect on total oil accumulation or significant change in the distribution of PC or TAG molecular species as revealed by MALDI-MS. However, biochemical experiments of oil accumulation in *B. napus* suggested that, because glycerol 3-phosphate levels were low^8^, endogenous GPAT activity was normally sufficient not to constrain TAG formation appreciably. Hence a lack of an observed increase in oil accumulation in GPAT-OE in this study may be due to the fact that GPAT activity does not exert significant flux control on TAG production.

In contrast to GPAT-OE lines, overexpression of the second Kennedy pathway enzyme, LPAT, caused an increase in seed TAG despite its low intrinsic flux control coefficient^24^. Moreover, we show here that LPAT overexpression changed the cellular distribution of PC and TAG molecular species. The MALDI-MS analysis showed that there was a general decrease in the unsaturation of TAG and, notably, of PC molecular species. Similar to the LPAT transgenics, the PDAT transgenics also showed significant alterations in the cellular distribution of molecular species of PC and TAG as well as a decrease in the unsaturation of these lipids. Although we did not evaluate the reason for the reduced unsaturation, it would be consistent with increased PDAT activity allowing less time for PC-mediated desaturation^34^. We note that total fatty acid content was reduced in the LPAT-OE and PDAT-OE lines but not in GPAT-OE at the mid-stage of seed development, which is the phase of rapid oil accumulation. This may in turn result in the altered lipid accumulation and distribution observed later in development in mature embryos, as shown by MALDI-MS analysis.

For the three transgenic lines, transcriptomic analysis revealed that LPAT-OE had the most DEGs, followed by GPAT-OE with the fewest DEGs detected in PDAT-OE, with substantially more up-regulated genes detected than down-regulated genes in all three transgenic lines. In oilseed rape, studies have shown that the final steps for TAG biosynthesis are catalysed mainly by the Kennedy pathway enzyme diacylglycerol acyltransferase (DGAT) rather than PDAT^6,28^. In that regard, it is perhaps not surprising that up-regulation of other enzymes involved in the Kennedy pathway (GPAT and LPAT) caused more changes in the DEGs detected by transcriptomic analysis than PDAT-OE.

Although the core genes encoding enzymes for oil accumulation showed no significant changes in the transgenic lines, there were notable alterations in the expression of other genes related to acyl-lipid metabolism. Several of these DEGs were common between different transgenics. For example, cysteine *S*-palmitoyltransferase (CPT), TRIGALACTOSYLDIACYLGLYCEROL2 (TGD2), malonyl-CoA decarboxylase (MCD), several ABC transporter G family members (ABCG) and a number of wax ester synthase/DGATs (WSD/DGAT) showed common alterations. CPT catalyses protein acylation and several isomers have been detected in plants^35^. TGD2 is involved in lipid transport between the ER and the chloroplast, while MCD has well-known functions in regulating fatty acid metabolism in bacteria and mammals^36,37^ where the decarboxylation reaction has been mainly examined as part of the polyketide synthase proteins^38^. ATP-binding cassette (ABC) transporters function in transmembrane transport and plant genomes encode a large number which have been divided into eight subfamilies. They can transport a variety of substrates, including many lipids^39^. WSD/DGAT is a bi-functional enzyme which has been reported in a wide variety of organisms^40^. Its primary function is for the formation of surface wax esters^41^ though it has been suggested that it could be responsible for making small amounts of TAG^42^.

The other DEGs identified in GPAT-OE include a gene encoding 4-coumarate-CoA ligase, which plays an important function in the biosynthesis of plant secondary compounds^43^. Two lipase genes were upregulated (GLIP3, Lipase 3) of which the GDSL esterase/lipase 3 plays a role in the esterification of lutein^44^ although its gene family is diverse with multifunctionality^45^. A second protein acyltransferase (PAT 13) was upregulated while acyl-CoA binding protein2 (*ACBP2*) was downregulated, though this expression change did not meet our strict DEG criteria.

LPAT-OE showed additional DEGs compared to the GPAT-OE line. In particular, there was an increase in the expression of *LEC1*, which encodes a transcription factor that promotes expression of several genes involved in fatty acid biosynthesis^46^. In addition, there were more ABC G family transporter genes and WSD/DGAT genes whose expression was altered. In addition, several DEGs encoded lipid transport proteins (LTPs), which are encoded by multigene families and have functions in many physiological processes^47,48^. A total of 63 putative non-specific LTP genes have been identified in *B. napus*^49^. Other DEGs which have functions in lipid transport are seipin-1^50,51^ and *TRIGALACTOSYLDIACYLGLYCEROL2* (TGD2)^52^, the latter of which showed up-regulation in all transgenic lines.

## Conclusions

Transgenic *B. napus* lines in which genes for key enzymes for TAG biosynthesis have been up-regulated showed differences in lipid accumulation, quality and distribution of PC and TAG molecular species, in addition to differential expression of genes involved in acyl-lipid metabolism and other molecular functions. These alterations could not be easily predicted based on our current knowledge of this important oil crop and emphasise the need for continuing work in the area. The observations reported here can form the foundation for such research.

## Materials and methods

### Plant materials

*Brassica napus* (oilseed rape) cv. DH12075 originally obtained from Agriculture and Agri-Food Canada, Saskatoon, Saskatchewan, Canada and homozygous LPAT-OE line TF2-14-85, GPAT9-OE line TF4-37-74, and PDAT1-OE line TF8-24-18 were provided by University of Durham. All transgenic lines contained single transgene insertions. These lines are referred to as WT, LPAT-OE, GPAT9-OE and PDAT1-OE in this work. Wild-type and transgenic seeds were germinated in pots containing soil/sand mix. Ten-day-old seedlings were transplanted individually into 8.7-inch pots. For data in Fig. 1, plants were grown in a greenhouse with a temperature of 23 °C and a light period (11–13 h) at School of Biological Sciences, the University of Hong Kong. Flowers were pollinated manually and tagged on the first day when flowers open. Siliques were collected at 38 days after pollination (DAP), representing the mid-stage of lipid accumulation in oilseed rape. For transcriptomics analysis, plants were grown at the School of Biosciences, Cardiff University, UK in a sand/soil mix in an environmentally controlled growth chamber at 22 °C under 16 h light/8 h dark regime. Siliques were collected at 27 DAP which representing the mid-stage of lipid accumulation under these growth conditions. For MALDI analysis, mature seeds cultivated in Cardiff under the conditions described above were used.

The *Arabidopsis thaliana PDAT1* sequence, At5g13640, was synthesized by Genscript, USA and cloned into pEntr1A-Napin-NosT between the *Brassica napus* Napin promoter and nos terminator sequences via *Nde*I and *Eco*RI sites. The Napin promoter-PDAT cassette was then transferred by Gateway LR Clonase II recombination into binary destination vector pRMH009, which contained the *nptII* gene expressed from the *nos* promoter, for selection of transformed material in tissue culture. The resultant final plasmid, pEW227-PDAT1, was verified by restriction mapping and sequencing the insert. The *Arabidopsis thaliana GPAT9* sequence, At5g60620, was cloned into pEntr1A-Napin-NosT between the *Brassica napus* Napin promoter and Nos terminator sequences via *Nde*I and *Eco*RI sites. The Napin promoter-GPAT cassette was then transferred by Gateway LR Clonase II recombination into binary destination vector pRMH009, which contained the *nptII* gene expressed from the Nos promoter, for selection of transformed material in tissue culture. The resultant final plasmid was verified by restriction mapping and sequencing the insert. The construct used to produce LPAT/LPAAT (*TmLPAT2*) overexpressing lines of *B. napus* has been described previously^24^.

For PDAT-OE, twenty-four independent T0 transgenic plants were regenerated from one transformation experiment and confirmed as transformed by PCR amplification of the *PDAT* gene plus T-DNA copy number determination by Southern blotting. Three homozygous lines containing a single copy of the transgene were characterized and one representative line (line TF8-24-18) was used for subsequent analyses. For LPAT-OE, twenty‐two T0 transgenic plants were regenerated from three independent transformation experiments and confirmed as transformed by PCR presence of the LPAAT gene of interest. The T‐DNA copy number was determined by qPCR assay, based on the nptII gene and the number of T‐DNA integration loci were determined by Southern blotting. Three homozygous lines with independent single-copy insertions of the transgene were identified and characterized, and one representative line (TF2-14-85) was used for subsequent analyses. For GPAT-OE, twenty-six independent T0 transgenic lines were generated and confirmed as transformed by PCR amplification of the *AtGPAT9* gene plus T-DNA copy number determination by Southern blotting. Three homozygous lines with independent single-copy insertions of the transgene were identified and characterized, and one representative line (TF4-37-74) was used for subsequent analyses.

Oilseed rape transformation was carried out as previously described^53^ with *Agrobacterium tumefaciens* AglI pEW227-PDAT1, AglI pBI121/NastLPAT, AglI pRMH009/GPAT9 transformation of DH12075 cotyledonary petioles. Transformed shoots were regenerated in the presence of 15 mg l^−1^ kanamycin. Rooted shoots were transferred to Jiffy-7 pellets (Jiffy Products, Kristiansand, Norway), acclimatised to growth chamber conditions and transferred to 12 cm pots containing Levington M2 compost with 5 g/l slow-release fertilizer as above. T_0_ plants were self-fertilised and T_1_ seed harvested from all lines.

All plant research, including the use of transgenic plants, was conducted in compliance with international and UK guidelines.

### Morphological analysis of Hong Kong-grown *Brassica* embryos at middle developmental stage

Due to the differences in light intensity and daylight length between the greenhouses in Hong Kong (11–13 h of natural light) and Cardiff (16 h with a light intensity of 250 µmol m^−2^ s^−1^)^28,31^, both wild-type and transgenic *B. napus* grew slower in Hong Kong than Cardiff. For Cardiff-grown wild-type *B. napus*, siliques were collected at 27 DAF, representing the mid-stage of lipid accumulation^28,31,54^). For Hong Kong-grown *B. napus*, 38 DAF was selected to represent the middle stage of lipid accumulation. The Hong Kong-grown wild-type and transgenic embryos at 38 DAF were dissected manually by a razor blade and photographed^31^. Seeds from ten siliques from each of six plants were harvested for measurements of mature seed weight.

### Fatty acid profiling

Twelve seeds from different siliques were collected at 38 DAF for each biological repeat. In total, six biological repeats were used. Fatty acid extraction was conducted as previously described^28,31^. Fatty acid methyl esters (FAMEs) were obtained by incubating seed samples in 1.2 ml of isopropanol at 70 °C for 30 min followed by incubation in 3 ml of 2.5% H_2_SO_4_ in methanol:toluene (2:1, v/v) at 70 °C for 2 h. Nonadecanoic acid (19:0) (Sigma) (20 µg) was added as an internal standard. 2 ml of 5% NaCl and 3 ml of hexane were mixed with the samples, and the upper phase was collected after brief centrifugation. Subsequently, another 3 ml of hexane was mixed with the samples, and the upper phase was collected. The combined hexane phase was dried by nitrogen gas. FAMEs were analysed by an Agilent GC–MS device (5973 inert mass spectrometer combined with 6890N gas chromatograph) equipped with an Agilent J&W DA-WAX capillary column (30 m × 0.25 mm × 0.25 µm). The oven temperature was 170 °C for 3 min, increased at 4 °C min^−1^ to 220 °C, and held for 15 min at 220 °C^31^. FAMEs were identified by comparing the retention time of peaks with the Supelco 37 Component FAME MIX standard (Sigma).

### MALDI-MS analysis

Dry seeds of *B. napus* were embedded in 10% gelatin, frozen for 16 h at − 80 °C and processed further as detailed^55^. Tissue sections (10 μm thickness) were imaged in a MALDI-LTQ-Orbitrap mass spectrometer (ThermoScientific). Set parameters were 12 μJ laser energy, 10 laser shots, 40 μm raster step size, collected over a m/z range of 700–1000 with the Orbitrap mass analyser set to a resolution of 60,000. Analysis used MS imaging software Metabolite Imager^56^ and MSiReader^57^. Molecular species of TAG were collected as [M + K]+ adducts while those of PC were imaged as the sum of [M + H]= and [M + K]+ adduct intensities. The molecular classes used a mass tolerance of 10 ppm for imaging and are presented as mol% on a colour scale from green (low) to red (high).

Calculation of average mol% for individual tissues summed the ion intensities of individual molecular species for each pixel where these represented laser spots and then normalising the total summed ion count for the PC or TAG lipid classes. Tissue areas evaluated were the embryonic axis, inner and outer cotyledons. Three replicate MS images were averaged and standard deviations plotted in bar charts for each genotype and the three tissue areas.

### RNA extraction

Siliques were harvested at 27 DAF. Embryos were manually dissected by a razor blade and stored immediately in liquid nitrogen before RNA extraction. Total RNA from embryos was extracted using RNeasy Plant Mini kit (Qiagen). On-column DNase digestion (Qiagen) was conducted on RNA samples to remove potential DNA contamination.

### RNA-seq analysis

Embryo RNA samples from selected three transgenic lines (GPAT9-OE, LPAT-OE and PDAT1-OE) and wild type with three biological replicates, were used for transcriptomics analysis on an Illumina NextSeq500 machine. Paired end sequencing (2 × 75 bp) strategy was used in the RNA seq analysis. The *B. napus* genome from ‘Darmor-*bzh*’was used as a reference genome^58^ RNAseq reads were quality filtered using Trimmomatic v. 0.38^59^ and resulting sequences were mapped with STAR v. 2.7.3^60^ to the Plants Ensembl reference Darmor-*bzh* genome v.5^58^. Optical duplicates were removed with Picard Tools (http://broadinstitute.github.io/picard/) and genomic features were recovered from the corresponding Darmor-*bzh* v.5 annotation. RNA-seq reads were counted using featureCounts^61^ and further analysed for differential gene expression using SARTools^62^ and DESeq2^63^. Gene orthology and gene set enrichment analysis (GSEA) were performed with g:Profiler^64^ against all known *Brassica napus* genes in Ensembl v.102 using a g:SCS threshold of 0.05. GSEA results were semantically summarised using REVIGO^65^ against the whole UniProt database.

## Supplementary Information

Supplementary Information.

Supplementary Table S2.
