# Supplementary material for: Transgenic manipulation of triacylglycerol biosynthetic enzymes in B. napus alters lipid-associated gene expression and lipid metabolism
Source: Sci Rep. 2022 Mar 1;12:3352. doi: 10.1038/s41598-022-07387-x (PMC8888550; doi:10.1038/s41598-022-07387-x)
Supplement: Supplementary file 1 — Supplementary Information. [file 41598_2022_7387_MOESM1_ESM.pdf]

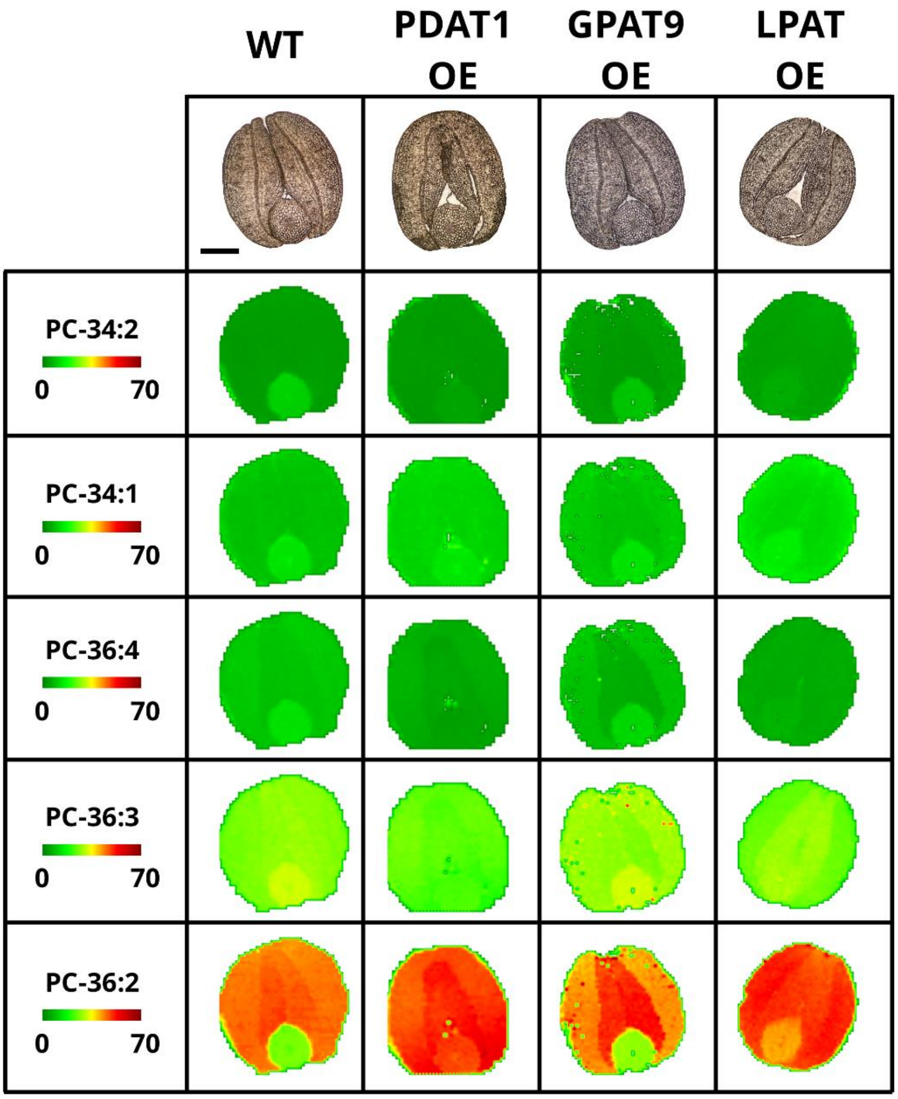

Supplemental Figure 1. MS imaging of PC in mature embryos from the WT and transgenic lines. MS images of selected PC molecular species for each genotype set at the same intensity value to show the relative abundance and distribution.

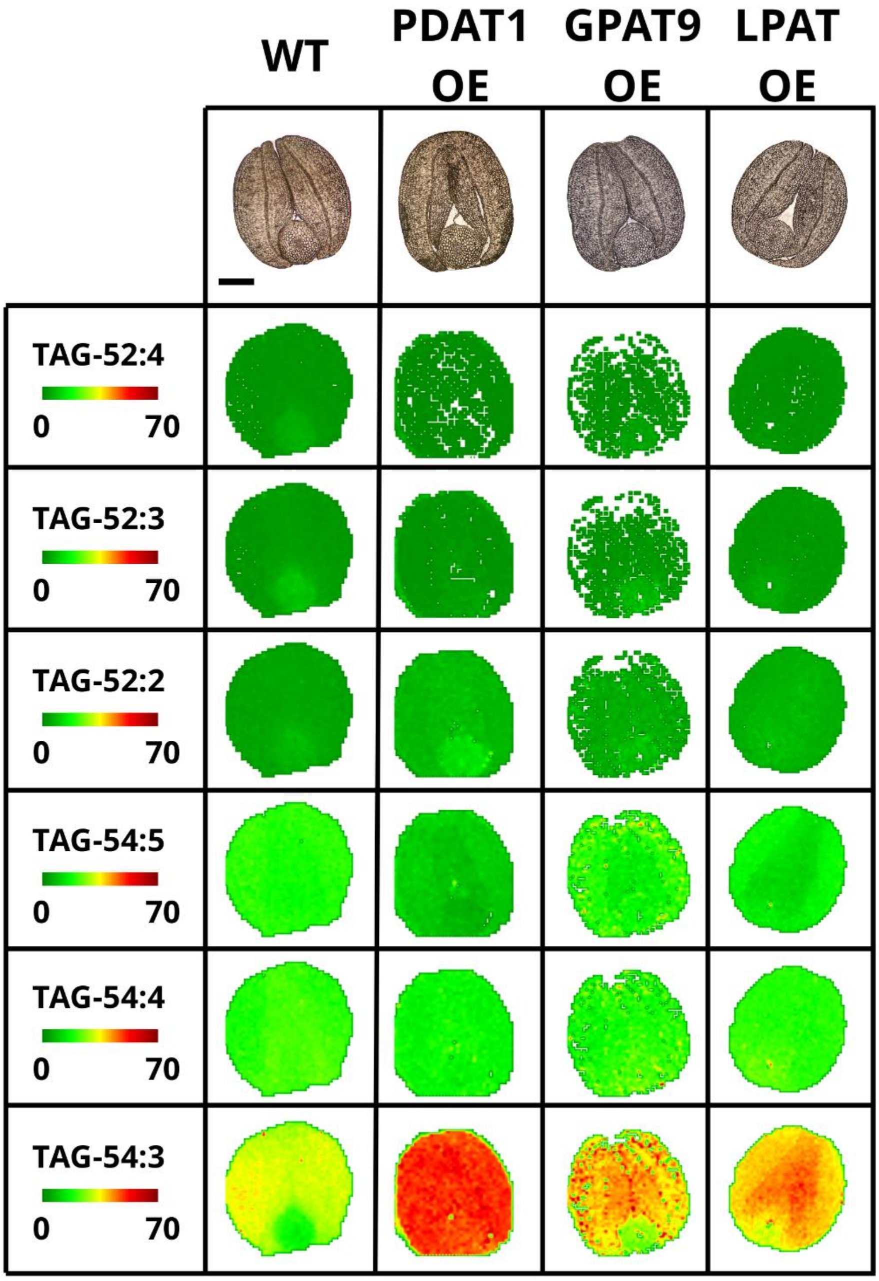

Supplemental Figure 2. MS imaging of TAG in mature embryos from the WT and transgenic lines. MS images of selected TAG molecular species for each genotype set at the same intensity value to show the relative abundance and distribution.

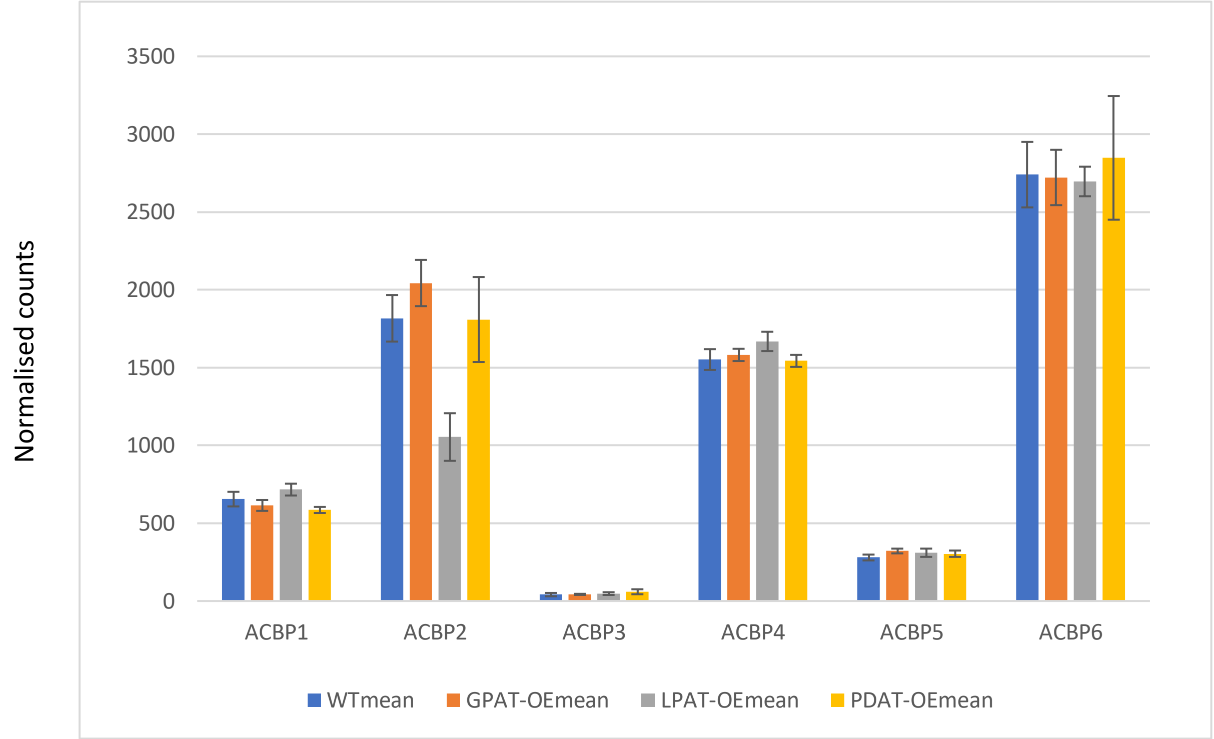

	WTmean	GPAT-OEmean	LPAT-OEmean	PDAT-OEmean
ACBP1	655	614	716	585
ACBP2	1817	2043	1054	1809
ACBP3	41	43	48	60
ACBP4	1552	1581	1668	1543
ACBP5	280	322	310	304
ACBP6	2740	2722	2696	2848

Supplementary Figure S3. Expression of *ACBP* genes in WT and transgenic lines by RNA-seq analysis. For each *ACBP*, the normalised counts represent the sum of the expression values for each differentially-spliced isoform.

Species	WT			GPAT-OE				LPAT-OE				PDAT-OE			
PC-34:3	**1.08**	+/-	0.06	**1.02**	+/-	0.10		**0.62**	+/-	0.09	**	**0.62**	+/-	0.09	**
PC-34:2	**4.33**	+/-	0.19	**4.91**	+/-	0.31	*	**3.91**	+/-	0.58		**3.24**	+/-	0.47	**
PC-34:1	**9.59**	+/-	0.39	**10.64**	+/-	0.47	*	**12.98**	+/-	0.72	**	**11.78**	+/-	0.39	**
PC-34:0	**0.41**	+/-	0.25	**0.80**	+/-	0.08	*	**0.36**	+/-	0.40		**0.39**	+/-	0.40	
PC-36:6	**0.08**	+/-	0.05	**0.05**	+/-	0.02		**0.02**	+/-	0.01		**0.01**	+/-	0.00	*
PC-36:5	**1.31**	+/-	0.27	**1.17**	+/-	0.11		**0.52**	+/-	0.11	**	**0.49**	+/-	0.10	**
PC-36:4	**9.06**	+/-	0.97	**8.57**	+/-	0.44		**5.50**	+/-	0.61	**	**5.05**	+/-	0.51	**
PC-36:3	**27.44**	+/-	0.44	**27.32**	+/-	0.57		**24.03**	+/-	2.34	*	**22.40**	+/-	1.52	**
PC-36:2	**45.80**	+/-	1.84	**42.40**	+/-	1.65	*	**51.34**	+/-	3.39	*	**54.27**	+/-	4.47	*
PC-36:1	**0.89**	+/-	1.01	**3.12**	+/-	0.43	**	**0.71**	+/-	0.83		**1.75**	+/-	1.75	
PC-36:0	**0.01**	+/-	0.00	**0.01**	+/-	0.00		**0.01**	+/-	0.01		**0.00**	+/-	0.00	

Species	WT			GPAT-OE				LPAT-OE				PDAT-OE			
TAG-50:4	**0.01**	+/-	0.00	**0.01**	+/-	0.01		**0.01**	+/-	0.00		**0.00**	+/-	0.00	
TAG-50:3	**0.02**	+/-	0.02	**0.05**	+/-	0.05		**0.02**	+/-	0.01		**0.01**	+/-	0.01	
TAG-50:2	**0.05**	+/-	0.06	**0.11**	+/-	0.08		**0.05**	+/-	0.02		**0.03**	+/-	0.02	
TAG-50:1	**0.04**	+/-	0.05	**0.06**	+/-	0.05		**0.09**	+/-	0.02		**0.07**	+/-	0.07	
TAG-52:7	**0.02**	+/-	0.02	**0.01**	+/-	0.01		**0.01**	+/-	0.00		**0.05**	+/-	0.08	
TAG-52:6	**0.07**	+/-	0.06	**0.09**	+/-	0.11		**0.09**	+/-	0.05		**0.11**	+/-	0.07	
TAG-52:5	**0.22**	+/-	0.20	**0.38**	+/-	0.27		**0.37**	+/-	0.18		**0.11**	+/-	0.03	
TAG-52:4	**1.87**	+/-	0.67	**2.51**	+/-	0.23		**1.90**	+/-	0.28		**1.05**	+/-	0.43	
TAG-52:3	**3.10**	+/-	0.60	**3.48**	+/-	0.75		**3.12**	+/-	0.29		**2.43**	+/-	0.64	
TAG-52:2	**5.90**	+/-	0.90	**6.16**	+/-	1.19		**7.05**	+/-	0.61		**7.54**	+/-	0.46	*
TAG-52:1	**0.17**	+/-	0.17	**0.32**	+/-	0.14		**0.21**	+/-	0.10		**0.32**	+/-	0.26	
TAG-54:9	**0.01**	+/-	0.01	**0.01**	+/-	0.02		**0.01**	+/-	0.00		**0.00**	+/-	0.01	
TAG-54:8	**0.11**	+/-	0.18	**0.09**	+/-	0.12		**0.04**	+/-	0.03		**0.01**	+/-	0.01	
TAG-54:7	**1.26**	+/-	1.21	**1.90**	+/-	0.64		**0.99**	+/-	0.45		**0.22**	+/-	0.17	
TAG-54:6	**4.39**	+/-	2.15	**4.84**	+/-	0.57		**3.46**	+/-	1.07		**1.62**	+/-	0.58	*
TAG-54:5	**16.12**	+/-	3.49	**17.41**	+/-	1.48		**14.00**	+/-	2.04		**10.33**	+/-	0.53	*
TAG-54:4	**20.54**	+/-	2.71	**19.10**	+/-	0.82		**19.29**	+/-	1.06		**17.56**	+/-	1.25	
TAG-54:3	**40.38**	+/-	8.56	**36.44**	+/-	1.79		**42.31**	+/-	4.63		**51.27**	+/-	4.37	
TAG-54:2	**2.39**	+/-	0.87	**3.43**	+/-	0.22		**1.83**	+/-	1.11		**2.67**	+/-	1.03	
TAG-54:1	**0.07**	+/-	0.03	**0.07**	+/-	0.05		**0.11**	+/-	0.04		**0.14**	+/-	0.09	
TAG-56:7	**0.01**	+/-	0.01	**0.03**	+/-	0.04		**0.01**	+/-	0.01		**0.01**	+/-	0.01	
TAG-56:6	**0.02**	+/-	0.02	**0.04**	+/-	0.05		**0.01**	+/-	0.01		**0.01**	+/-	0.00	
TAG-56:5	**0.21**	+/-	0.20	**0.35**	+/-	0.22		**0.36**	+/-	0.13		**0.14**	+/-	0.13	
TAG-56:4	**0.61**	+/-	0.25	**0.76**	+/-	0.18		**1.01**	+/-	0.15	*	**0.58**	+/-	0.29	
TAG-56:3	**1.74**	+/-	0.66	**1.83**	+/-	0.10		**2.43**	+/-	0.32		**2.40**	+/-	0.51	
TAG-56:2	**0.67**	+/-	0.48	**0.51**	+/-	0.05		**1.23**	+/-	0.36		**1.30**	+/-	0.44	
TAG-56:1	**0.01**	+/-	0.01	**0.01**	+/-	0.01		**0.02**	+/-	0.01		**0.03**	+/-	0.02	

Table S1. MALDI-MS quantification (mol%) of PC and TAG molecular species in mature embryos of WT, GPAT-OE, LPAT-OE and PDAT-OE lines. Means +/- S.D. (n=4) are shown. * indicates significance in *t*-test (p<0.05). ** indicates significance in *t*-test (p<0.01).
